# Transformation of Cu_2_O into Metallic Copper within Matrix of Carboxylic Cation Exchangers: Synthesis and Thermogravimetric Studies of Novel Composite Materials

**DOI:** 10.3390/ma17163893

**Published:** 2024-08-06

**Authors:** Elżbieta Kociołek-Balawejder, Katarzyna Winiarska, Juliusz Winiarski, Igor Mucha

**Affiliations:** 1 Department of Industrial Chemistry, Wroclaw University of Economics and Business, Komandorska 118/120, 53-345 Wrocław, Poland; elzbieta.kociolek-balawejder@ue.wroc.pl; 2 Department of Inorganic Chemistry, Wroclaw University of Economics and Business, Komandorska 118/120, 53-345 Wrocław, Poland; katarzyna.winiarska@ue.wroc.pl; 3 Groups of Surface Technology, Department of Advanced Material Technologies, Wroclaw University of Science and Technology, Wybrzeże Wyspiańskiego 27, 50-370 Wrocław, Poland; juliusz.winiarski@pwr.edu.pl; 4 Department of Basic Chemical Sciences, Wroclaw Medical University, Borowska 211 A, 50-556 Wrocław, Poland

**Keywords:** metallic copper, cuprous oxide, carboxylic cation exchanger, hybrid ion exchanger, composite materials, thermal analysis

## Abstract

In order to systematize and expand knowledge about copper-containing composite materials as hybrid ion exchangers, in this study, fine metallic copper particles were dispersed within the matrix of a carboxyl cation exchanger (CCE) with a macroporous and gel-type structure thanks to the reduction of Cu_2_O particles precipitated within the matrix earlier. It was possible to introduce as much as 22.0 wt% Cu^0^ into a gel-type polymeric carrier (G/H#Cu) when an ascorbic acid solution was used to act as a reducer of Cu_2_O and a reagent transforming the functional groups from Na^+^ into the H^+^ form. The extremely high shrinkage of the porous skeleton containing –COOH groups (in a wet and also dry state) and its limited affinity for water protected the copper from oxidation without the use of special conditions. When macroporous CCE was used as a host material, the composite material (M/H#Cu) contained 18.5 wt% Cu, and copper particles were identified inside the resin beads, but not on their surface where Cu^2+^ ions appeared during drying. Thermal analysis in an air atmosphere and under N_2_ showed that dispersing metallic copper within the resin matrix accelerated its decomposition in both media, whereby M/H#Cu decomposed faster than G/H#Cu. It was found that G/H#Cu contained 6.0% bounded water, less than M/H#Cu (7.5%), and that the solid residue after combustion of G/H#Cu and M/H#Cu was CuO (26.28% and 22.80%), while after pyrolysis the solid residue (39.35% and 26.23%) was a mixture of carbon (50%) and metallic copper (50%). The presented composite materials thanks to the antimicrobial, catalytic, reducing, deoxygenating and hydrophobic properties of metallic copper can be used for point-of-use and column water/wastewater treatment systems.

## 1. Introduction

Copper, alongside iron and aluminum, is one of the most useful metals on the planet. It exhibits exceptional physical properties, such as plasticity and ductility, excellent thermal and electrical conductivity and corrosion resistance. Traditional areas of copper use are the electrical, energy, telecommunications and construction sectors, and the most important copper products are electrical wiring and equipment. Over the years, the biostatic properties of copper have been used for sterilizing water [1,2]. Its antifungal action has been used for many years in agriculture and crop protection [3]. Currently (in May 2024), for the first time, the price of copper has exceeded 11,000 USD/t, and its level is one of the parameters determining trends in the global economy. In recent years, the growing demand for copper has been related to the progress in renewable energy sources, the production of electric cars, the expansion of AI data centers and the development of modern defense systems. Crucial projects in the global economy related to the energy transition are based on investments with a key share of copper. In view of the high demand for copper, it is worth emphasizing that it is a versatile material that can be processed many times without a loss of quality. From used products, scrap and waste, it is relatively easy to obtain so-called blister copper, containing 98.5 wt% Cu.

The development of nanotechnology in the last two decades has broadened the scope of the use of copper in the form of ultrafine particles. Copper nanoparticles (CuNPs), which exhibit fascinating physicochemical and biological properties, have found applications which are not achievable in the case of copper in bulk form in the fields of medicine [4,5] and the environment [6], amongst others. The antimicrobial properties of CuNPs are used in healthcare [7,8] and also in water treatment [9] and food packaging, as well as in antifouling coatings due to their relevance as media through which diseases are easily spread [10]. Unlike antibiotics, which are administered to treat diseases and infection in patients, CuNPs provide an opportunity to limit microbial growth prior to human infection. CuNPs demonstrate a sensing mechanism in relation to Hg^2+^, Pb^2+^, NO_2_^−^, S^2−^, I^−^, and 2,4,6-trinitrotoluene, useful in the analysis of trace water contaminants [11]. They activate sulphites [12,13] and peroxysulphates [14,15] for the elimination of organic pollutants in aqueous environments. The photoredox/thermocatalytic technology in which the metallic copper is introduced into in the catalytic system addresses priority issues such as the production of high value-added carbon fuel, splitting of water for H_2_ production, reduction of CO_2_ (CH_4_ production), organic matter transformation, and environmental remediation such as the degradation of pollutants in sewage [16,17,18]. The weakness of CuNPs is their susceptibility to oxidation. This is prevented by oxygen-free preparation in nonaqueous media and surface passivation using protecting reagents [19,20].

The extremely small size of the nanoparticles causes various difficulties and limitations during their preparation, storage and handling [21,22,23]; however, these can be overcome by coupling them with support materials [24,25]. To stabilize them and prevent aggregation/agglomeration, hybrid ion exchangers (HIXs) have been developed, in which the polymeric supports for dispersed nano-/submicroparticles are synthetic ion exchangers, more precisely commercial cation exchangers [26] and anion exchangers [27,28] used in large-scale water treatment processes. HIXs, like ion exchangers, show a high accessible surface area and fast kinetics, and due to the physical form of the spherical polymeric beads, they can be used in dynamic conditions in fixed-bed column systems (nanoparticles alone are used only in batch processes, which causes various environmental and technical problems). The most common HIXs contain hydrated polyvalent metal oxides/hydrated oxides/oxyhydroxides (Fe(III), Mn(IV), Zr(IV), Al(III), Ti(IV) embedded in a matrix of strongly basic anion exchangers, and due to the presence of anion exchange functional groups, they effectively and selectively remove various harmful oxyanions from water (according to the Donnan membrane effect) [29].

On the basis of strongly basic anion exchangers, HIXs with dispersed CuO, CuO/FeO(OH), Cu_2_O, Cu_4_O_3_, Cu(OH)_2_, Cu_2_(OH)_3_Cl, Cu_4_(OH)_6_SO_4_, and also fine Cu^0^ particles (3.5–12.5 wt% Cu) have been synthesized recently [30]. Some of them have been used in water treatment processes in which their multifunctionality was essential, i.e., the ability to act in many ways, as an adsorbent, oxidant, biocide and catalyst. Thermogravimetric studies on their high-temperature behavior were the first comprehensive studies into HIXs [31,32]. The experiments carried out under air and under N_2_ showed the effect of the oxidation state of the copper atom in the deposit on the decomposition of the polymeric phase (styrene/divinylbenzene copolymers with trimethylammonium functional groups). It has been found that two phases of these materials, the inorganic phase and polymeric phase, behave differently at high temperatures and also react with each other. Inorganic deposits accelerated resin decomposition, lowered the process end temperature, and affected the amount and composition of the post-pyrolysis residue (metallic copper and carbon in different proportions). During pyrolysis, the transforming polymeric phase reduced the inorganic deposit, whereby a larger part of it condensed into products with large non-volatile particles than in the case of the pyrolysis of pure resin. The oxidation state of the copper atom in the deposit had an increasing effect on the amount of the formed carbon [32]. It was also found that the oxidation state of copper-containing particles (CuO, Cu_2_O, Cu^0^) affected the textural properties of the polymeric phase, endowing it with a tighter structure, limiting the porosity and reducing the affinity for water [33]. The last property was particularly evident in the case of HIXs with Cu^0^ deposits containing anomalously little surface-bound/hygroscopic water, with rare amounts as low as around 4.0%. The Cu^0^ in the polymer matrix was a much stronger water repellent compared to Cu_2_O and CuO [31,32].

Recently, we undertook research on the thermal decomposition of HIXs containing CuO and Cu_2_O dispersed within the matrix of another type of ion exchanger, namely carboxylic cation exchangers (CCEs), acrylic acid/divinylbenzene copolymers [34,35]. It turned out that in materials containing carboxylic groups, thermal behavior was significantly influenced not only by the type of inorganic deposit (CuO or Cu_2_O) but also by the ionic form of these groups. For example, Cu_2_O-doped CCE with carboxylic groups in the H^+^ form decomposed faster than pure resin at below 200 °C, but Cu_2_O-doped CCE with carboxylic groups in the Na^+^ form was durable up to 450 °C [35]. CuO-doped CCE with carboxylic groups in the Cu^2+^ form decomposed much faster than pure resin and rarely during pyrolysis; a solid residue was formed of a mass that represented as much as 50 wt% of the sample starting mass, consisting mainly of metallic copper (up to 80%), the creation of which was due to the reduced division into CuO of the products of polymeric matter thermal decomposition [34]. It turned out that despite its high oxygen content (about 35.0 wt%), CCE was an effective reducer of CuO in the pyrolysis process, while the reduction of Cu_2_O to Cu^0^ in the case of Cu_2_O-doped CCE occurred only under certain conditions. In this study, this intriguing research was supplemented by a missing component, namely metallic copper (zero valent copper, ZVC) containing a carboxylic cation exchanger. Literature reports about these types of composites are scarce and concern the electrodeposition of copper into different ion exchangers [36,37].

To embed ultrafine metallic copper particles inside CCEs, previously prepared composite materials were used, namely Cu_2_O-doped CCEs [35]. We took into account that the chemical reduction of Cu_2_O is the shortest path to ZVC given the following sequence of transformations starting from cupric salts: Cu^2+^ → Cu(OH)_2_ → CuO → Cu_2_O → Cu^0^. Ascorbic acid was selected to transform Cu_2_O into Cu^0^, with the formation of dehydroascorbic acid as the two-electron transfer oxidation product. Ascorbic acid, a weak acid with two dissociation constants (K_a1_ = 1.0 × 10^−4^ and 1.6 × 10^−10^) and a green, mild reducing agent, is widely used in the synthesis of advanced nanomaterials [38,39,40,41]. Thus, the aim of this study was to obtain metallic copper containing carboxylic cation exchangers of the gel-type and with a macroporous structure, by the reduction of Cu_2_O particles previously deposited in polymeric supports, and then to examine their thermal behavior by thermal analysis. More precisely, we wanted to check (a) whether Cu_2_O precipitated in the resin phase can be quantitatively converted into Cu^0^ in mild conditions; (b) whether the course of the reaction is influenced by a pH < 7 (ascorbic acid vs. sodium ascorbate); (c) whether the type of supporting polymer structure (macroporous or gel-type) and the extraordinary ability of CCEs to change the volume depending on the ionic form of the functional groups influence the stability and distribution of the deposited particles; (d) whether fine metallic copper particles affect the thermal decomposition of CCE under air and under nitrogen. This research is expected to lead to unique and advanced composite materials with antimicrobial, catalytic, reducing, deoxygenating and hydrophobic properties for point-of-use and column treatment systems.

## 2. Materials and Methods

### 2.1. Materials

The polymer supports for fine Cu^0^ particles were Amberlite IRC86 (gel-type structure) and Amberlite IRC50 (macroporous), commercially available carboxylic cation exchangers with functional groups in the H^+^ form (M/H, G/H) (Fluka Chemika, Honeywell, NC, USA). All the chemicals used in the study, including CuSO_4_·5H_2_O (Chempur, Piekary Śląskie, Poland), ascorbic acid(+) (POCh, Gliwice, Poland), ammonia solution 25%, HCl 35% and NaOH (PPH Stanlab, Lublin, Poland), and cuprizone 98% (bis(cyclohexanone)oxaldihydrazone) (Alfa Aesar, at present Thermo Fisher Scientific, Ward Hill, MA, USA) were of analytical grade. All the solutions were prepared using deionized water.

### 2.2. Synthesis of CCEs with Metallic Copper Particles

All transformations were carried out in ambient conditions. First, G/H, M/H, ~10.0 g (moisture content approx. 45%) was placed in an Erlenmeyer flask and reacted with an excess of 1.0 M NaOH for 24 h. After this, the resin was moved to a glass column (diameter 1.13 cm) and 1.0 M NaOH was passed through the column until the NaOH concentration in the effluent equaled the concentration of the influent, after which the resin bed was rinsed with deionized water. In this way, resin samples were obtained with functional groups in the Na^+^ form (marked G/Na and M/Na). Analogously, in order to transform the functional groups in the Cu^2+^ form, 0.1 M CuSO_4_ was passed through the column, followed by deionized water. In this way, resin samples were obtained marked G/Cu and M/Cu. Two subsequent transformations were carried out in a bath regime. To obtain Cu_2_O-loaded resins, 2.0 g G/Cu, M/Cu (dried at 40 °C for 24 h in a dryer chamber) was reacted in an Erlenmeyer flask with 100 cm^3^ 0.5 M ascorbic acid in 2.0 M NaOH. After 24 h of shaking, the samples were drained and washed with deionized water, obtaining G/Na#Cu_2_O, M/Na#Cu_2_O. To obtain metallic copper-loaded resins, moist G/Na#Cu_2_O, M/Na#Cu_2_O (filtered off under vacuum) was treated in an Erlenmeyer flask with 100 cm^3^ 1.0 M ascorbic acid in water alone or in 1.0 M NaOH. The reagents were shaken for 24 h. After that, the filtered and washed products were dried at 35 °C in a dryer chamber. The Cu content in G/Cu, M/Cu (after treatment with 2.0 M HCl) and in G/Na#Cu_2_O, M/Na#Cu_2_O (after Cu_2_O load dissolution in a concentrated ammonia solution) was determined by spectrophotometric analysis of the obtained solutions using the cuprizone method.

### 2.3. X-ray Powder Diffraction Analysis

The X-ray powder diffraction patterns were measured on a Bruker D2 Phaser diffractometer (Bruker AXS, Karlsruhe, Germany) operating in Bragg–Brentano (θ/2θ) geometry. The device was equipped with a LINXEYE silicon strip detector and copper anode (Cu-Kα). The X-ray tube operated at 30 kV and 10 mA. The samples were previously ground in an agate mortar and placed into shallow-cavity zero background holders as flat pressed powders. X-ray powder diffractograms of each sample were recorded at room temperature in the same scanning range 5° ≤ (2θ) *≤* 70°, with a step size of 0.02° (2θ) and a count time of 0.2 s per step. The diffractometer optic was a Soller slit module with 2.5°, 0.2 mm divergence slit, 0.5 mm anti-scatter slit and a nickel filter. To interpret the data and compare the obtained XRD patterns, Measurement Suite and Diffrac.EVA v.3.2 (Bruker AXS, Karlsruhe, Germany) software was used.

### 2.4. Attenuated Total Reflectance–Fourier Transform Infrared Spectroscopy (ATR-FTIR)

Fourier-transform infrared spectra of the samples were collected using a Nicolet 380 FTIR spectrometer (Thermo Scientific, Waltham, MA, USA) equipped with an attenuated total reflectance (ATR) accessory component. Spectra were obtained in the range 400 cm^−1^ to 4000 cm^−1^ at a resolution of 4 cm^−1^ averaging 64 scans. Measurements for each sample were replicated three times to verify their uniformity. Each spectral acquisition was made with background subtraction, and all spectra were normalized. OMNIC software (v. 9) supplied from the manufacturer of the spectrometer was used for spectral data acquisition and analysis.

### 2.5. Scanning Electron Microscopy Analysis (SEM)

The morphology of the surface and cross-sections of HIXs (G/H#Cu, M/H#Cu) were analyzed using a Quanta 250 (FEI, Hillsboro, OR, USA) scanning electron microscope equipped with an Octane Elect Plus SDD microanalyzer (EDAX, Mahwah, NJ, USA) in SE and BSE modes at 25 kV. The distribution of elements across the samples was investigated on their cross-sections at 15 kV. Therefore, the modified carboxylic cation exchangers were previously embedded in an epoxy resin, polished (finally with a 0.25 μm polycrystalline diamond suspension), rinsed and dried in a vacuum desiccator until required. Sample preparation for SEM included sputtering the specimens with a thin layer of carbon (~8 nm).

### 2.6. Thermogravimetric Analysis

The thermogravimetric analysis (TG) and derivative thermogravimetry (DTG) were performed using a TG 209 F1 Libra thermogravimetric analyser (Netzsch, Selb, Germany). About 10 mg of sample was placed in an alumina crucible (150 μL) and heated from 25 °C up to 950 °C at a heating rate of 10 °C/min. The pyrolysis study was carried out using nitrogen (flow rate 30 mL/min), while dry air was used for combustion (flow rate 30 mL/min). The measurements were repeated multiple times under the same conditions to confirm the reproducibility and authenticity of the generated data. The TG and DTG curves, recorded with a ±1.5 °C precision, were analyzed using Netzsch Proteus 7.1.0 software (Selb, Germany).

## 3. Results and Discussion

### 3.1. Metallic Copper Deposition within G/H, M/H to Obtain G/H#Cu, M/H#Cu

Based on the effective immobilization of metallic copper within a matrix of strongly basic anion exchangers [42], we expanded the scope of the work by using, as host materials, ion exchangers of a different type, with different skeletons and different functional groups, namely carboxylic cation exchangers. The essence of the method was to embed Cu_2_O particles within the matrix of the resin and then reduce them to Cu^0^ in such a way that the transformed inorganic deposit quantitatively remained in the resin phase. As host materials, carboxylic cation exchangers have strengths, but also some special features and limitations. CCEs have the highest ion exchange capacity of all commercial synthetic ion exchangers (almost 11.0 meq/g), which is 3–4 times greater compared to sulfonic cation exchangers and all types of anion exchanges. This provides the opportunity to obtain HIXs rich in inorganic matter, because the first stage of the synthesis of this type of material is an ion exchange reaction involving ion exchange functional groups. It should be emphasized that among all types of ion exchangers, CCEs are characterized by an extraordinary tendency to change volume—when the carboxylic groups are transformed to the Na^+^ form, the resin swells strongly, and when the functional groups are transformed to the H^+^ form, the resin shrinks strongly (according to the manufacturer’s data, for carboxylic cation exchangers the maximum reversible swelling H^+^ → Na^+^ is 80–100%).

**Scheme 1:**
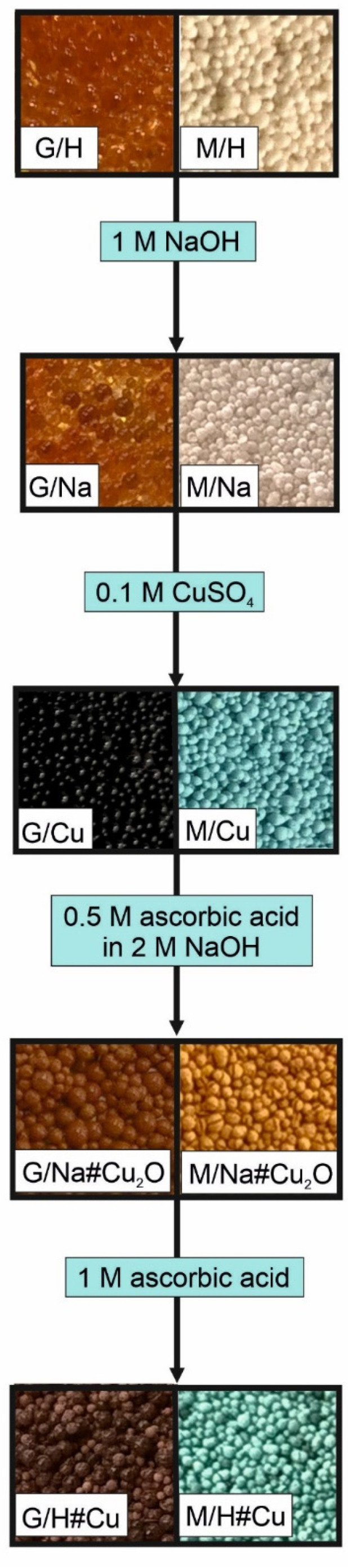
Carboxylic cation exchangers’ (CCEs) transformation steps for the embedding of metallic copper within their matrix. Photographs of examined materials based on a gel-type CCE (G)—left column—and on a macroporous CCE (M)—right column.

To introduce metallic copper into the matrix of carboxylic cation exchangers, a four-step transformation was needed. Scheme 1 shows photographs of two starting resins and the subsequent products derived from them. The ion exchange reactions (1) and (2) were carried out with the column method, and the redox reactions (3) and (4) were carried out with the batch method:

(1) $$\left[P\right]-COOH+NaOH\to \left[P\right]-COONa+H_{2}O$$

(2) $$2\left[P\right]-COONa+CuSO_{4}\to (\left[P\right]-COO{)}_{2}Cu+{Na}_{2}SO_{4}$$

(3) $$(\left[P\right]-COO{)}_{2}Cu\overset{\begin{array}{c} ascorbic\;acid\\ pH>7 \end{array}}{\to }\{2\left[P\right]-COONa\}\#Cu_{2}O$$

(4) $$\left[P\right]-COONa\#{Cu}_{2}O\overset{\begin{array}{c} \;\\ ascorbic\;acid\\ pH<7 \end{array}}{\to }\left[P\right]-COOH\#Cu$$

where [P] stands for polymeric matrix and # stands for deposited within polymeric matrix.

It was not possible to transform the carboxylic groups from H^+^ in the Cu^2+^ form in one step using a CuSO_4_ solution. Carboxylic groups show some characteristic features: (a) in the H^+^ form they do not split neutral salts, (b) the degree of ionization corresponds to that of acetic acid, (c) the effective pH is in the region of 6–14, (d) a characteristic property is high selectivity to H^+^, and (e) on changing the resin H^+^ form for another ionic form, great volume changes occur. Taking these facts into account, first the carboxylic groups were transformed into the Na^+^ form using a NaOH solution. In order to carry out ion exchange reaction (1) efficiently and quantitatively, it was performed in two stages, first batch wise, and then in the column (when the resin was immediately placed in the column, it swelled so strongly that the NaOH solution stopped flowing through the bed). After reaction (2), G/Cu and M/Cu (Table 1) were obtained containing as much as 22.0 wt% Cu.

Transformations (3) and (4) were carried out under batch conditions to ensure the homogeneity of the obtained composite materials. In both reactions, the reducer was ascorbic acid. When G/Cu and M/Cu were reduced with ascorbic acid in an alkaline medium, Cu_2_O precipitated within the resin matrix (3). Table 1 shows that the somewhat lower copper content in G/Na#Cu_2_O and M/Na#Cu_2_O compared to G/Cu and M/Cu was due to a difference in the bounded/hygroscopic water content, as the carboxylic groups in the Na^+^ form are highly hydrated. During reaction (3), with the use of M/Cu, some Cu_2_O precipitate passed into the aqueous phase, resulting in a lower copper content in M/Na#Cu_2_O compared to G/Na#Cu_2_O.

**Figure 1:**
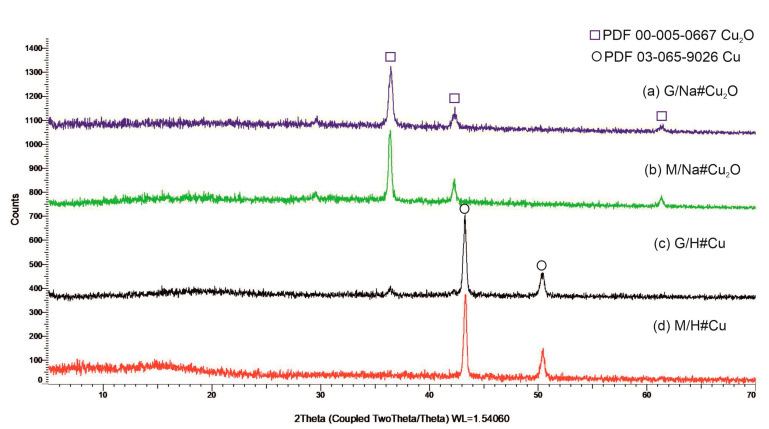
XRD patterns after reaction of G/Na#Cu_2_O and M/Na#Cu_2_O with sodium ascorbate (a,b) and with ascorbic acid (c,d).

In order to obtain metallic copper containing CCEs, Cu_2_O was reduced to Cu^0^ using ascorbic acid, but under different conditions than before. It was reported in the literature that the reduction of Cu^2+^ to Cu^0^ occurs at pH < 7, and that depending on the pH value in this range, differences are observed in the reaction rate and morphology of the CuNPs that are formed [38,39,40,41]. In this study, two reaction solutions with pH < 7 were used, a solution of ascorbic acid alone (with pH~2.0) and a solution of ascorbic acid neutralized with an equimolar amount of NaOH (with pH~6.0, sodium ascorbate). Previously, both solutions have been shown to be effective during the reduction of Cu_2_O deposited within the matrix of strongly basic anion exchangers [42]. In this study, the reduction of Cu_2_O to Cu^0^ was provided only by one of the solutions, namely the solution of ascorbic acid alone. When G/Na#Cu_2_O and M/Na#Cu_2_O were treated with the solution of sodium ascorbate, no reaction occurred. After the reaction, Cu_2_O was identified in both samples (Figure 1a,b). The intense peaks at about 30, 36, 42 and 61.5° 2θ correspond well to diffractogram No. 00-005-0667 from the PDF database. In this case, the samples are single-phase because there are no other peaks in the diffractogram that could be attributed to, for example, the CuO or Cu phase. The lack of reduction can be explained by the lack of ion exchange reaction between the –COONa groups and Na^+^ ions in the aqueous phase; therefore, ascorbate ions were not drawn inside the CCE. It turned out that the same low molecular reagents, Cu_2_O and sodium ascorbate, surrounded by different porous host materials (an anion exchanger with positively changed functional groups or a cation exchanger with negatively changed functional groups), react with each other or do not react. In this case, this was influenced by the inappropriate ionic form of carboxylic groups.

When the reaction medium was a solution of ascorbic acid alone, reduction of Cu_2_O to Cu^0^ occurred (Figure 1c,d). A small peak at 36° 2θ for a gel-type matrix sample (G/H#Cu) indicates that the reduction to metallic copper was not complete in the entire volume of the sample. The reason may be due to the compact structure of the sample, which makes access for the reducing agent difficult. The sample obtained from the HIX with a macroporous structure (M/H#Cu) is single-phase. Only peaks characteristic for Cu^0^ are visible, which correspond to the peaks from the PDF database (No. 03-065-9026). The different appearance of dry G/H#Cu and M/H#Cu (Scheme 1, the two photos at the bottom) is surprising, because brown G/H#Cu has a color resembling copper, while M/H#Cu is unexpectedly blue (it looks similar to M/Cu, Scheme 1). After reaction (4) and subsequent washing with deionized water and vacuum filtering, both samples were burgundy-brown. During drying in a drying chamber at 35 °C, M/H#Cu changed color to blue. Taking into account the results of the XRD analysis (Figure 1d) indicating the presence of one crystalline ingredient in the inorganic phase, namely metallic copper, it can be thought that due to the tendency of copper to oxidize in the presence of moisture, a reaction (5) occurred on the surface of the resin beads with the formation of blue-colored Cu^2+^ ions bounded by carboxylic groups:

2Cu + O_2_ + 2H_2_O → 2Cu^2+^ + 4OH^−^ (5)

**Figure 2:**
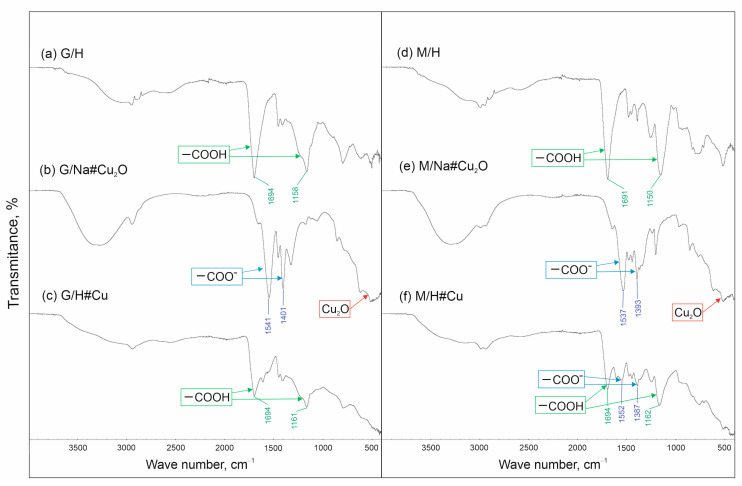
FTIR spectra of examined samples.

Figure 2 shows the FTIR spectra of six samples: both starting CCEs (G/H, M/H) and four HIXs (G/Na#Cu_2_O, M/Na#Cu_2_O, G/H#Cu and M/H#Cu). The spectra of G/H, M/H and also G/H#Cu (Figure 2a,c,d) contain peaks that can be assigned to undissociated carboxylic groups, while the spectra of G/Na#Cu_2_O and M/Na#Cu_2_O (Figure 2b,e) contain peaks derived from dissociated carboxylic groups (in Na^+^ form). The spectrum of M/H#Cu (Figure 2f) contains peaks of both types, which may indicate the presence of carboxylic groups partially in the Cu^2+^ form and partially as undissociated. The relatively limited occurrence of carboxylic groups in the ionic form in M/H#Cu provides a characteristic peak at about 3350 cm^−1^ indicative of adsorbed water. It can be observed that after reduction of Cu_2_O to Cu^0^, the area of this broad peak significantly decreases, which indicates that M/H#Cu contained less water than M/Na#Cu_2_O and was therefore dominated by undissociated carboxylic groups. Analyzing the FTIR spectra of four HIXs to identify the inorganic phase, one can see the characteristic peak of Cu(I)-O stretching vibrations at 650–550 cm^−1^ in the spectra of G/Na#Cu and M/Na#Cu (Figure 2b,e). The reduction of Cu_2_O to Cu^0^ was confirmed in the spectra of G/H#Cu and M/H#Cu (Figure 2c,f) by the disappearance of Cu-O bands in the far IR region (metallic copper is inactive in IR analysis).

**Figure 3:**
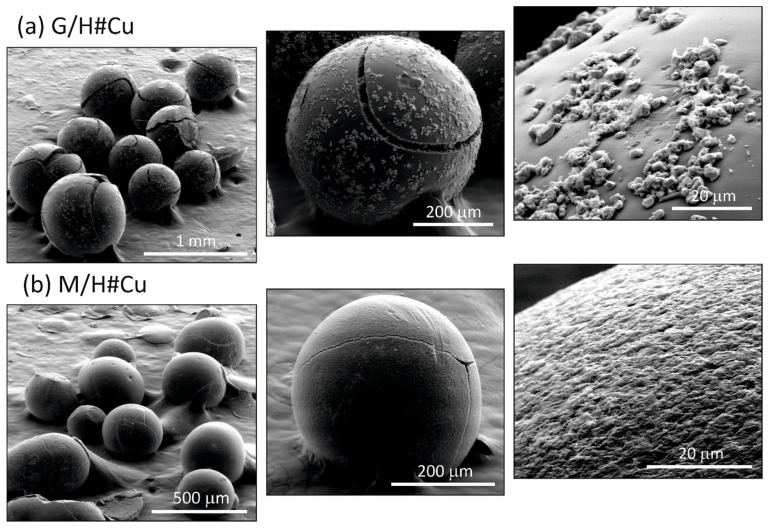
Morphology of the surface of G/H#Cu (**a**) and M/H#Cu (**b**).

**Figure 4:**
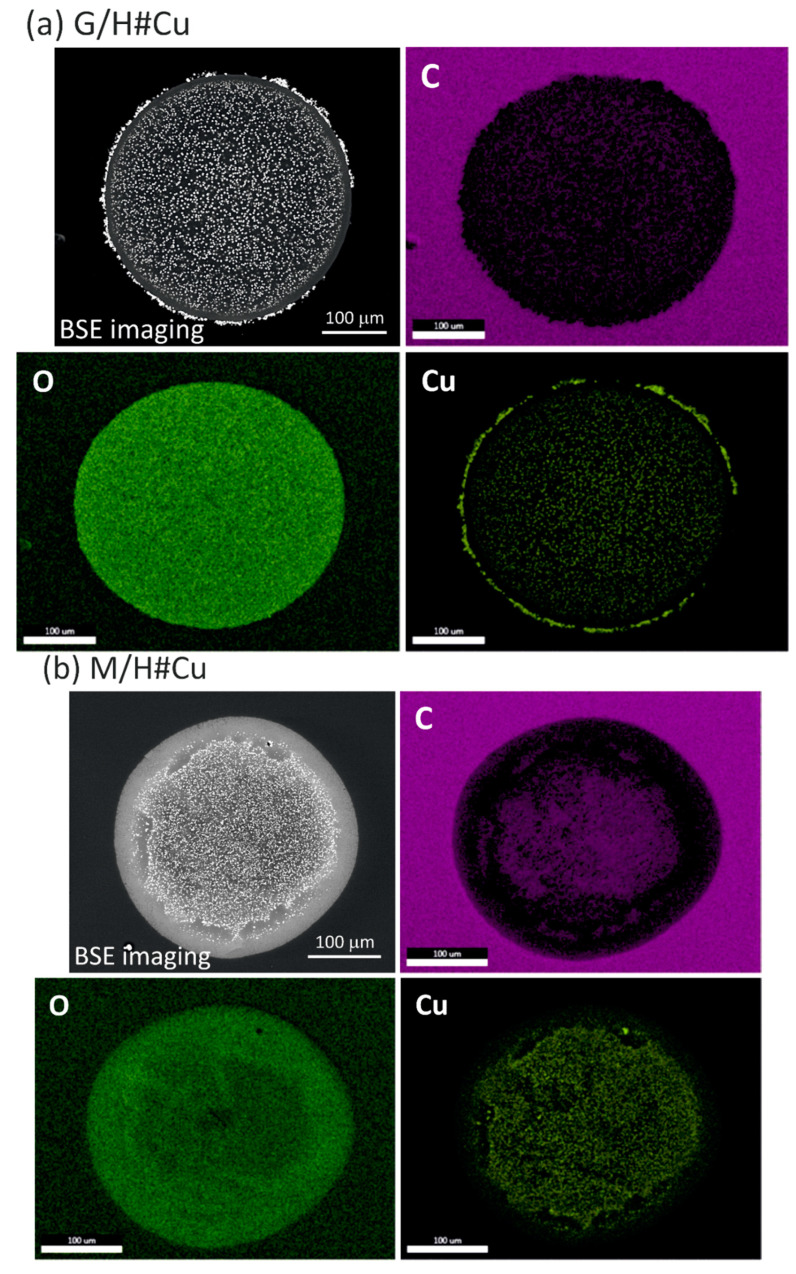
Cross-sections observed in a backscattered mode of SEM and corresponding mapping of the elements for G/H#Cu (**a**) and M/H#Cu (**b**).

SEM analysis revealed that the surfaces of both cation exchangers differ in morphology (Figure 3) and copper distribution (Figure 4). The sample obtained from the gel-type cation exchanger (Figure 3a) has an obviously smooth surface with numerous visible clusters of copper up to several micrometers in size. In turn, M/H#Cu (Figure 3b) has a wavy, developed surface with visible macropores. However, there are no larger crystallites on the surface. Both tested samples are characterized by visible cracks, which are much larger in the case of the gel-type cation exchanger. EDX analysis performed on cross-sections of the G/H#Cu and M/H#Cu samples allowed us to determine changes in the distribution of copper in the volume of the HIXs. The G/H#Cu sample has a core–shell structure in which copper is uniformly dispersed in the core (Figure 4a). The shell is a polymer matrix on the surface of which Cu^0^ clusters are formed. Due to the content of carboxylic functional groups, oxygen and carbon are visible as elements evenly distributed in the bulk of the tested sample. However, in the case of the macroporous cation exchanger (Figure 4b), copper is visible only in the interior, while the near-surface area is poor in Cu^0^. Moreover, inside the M/H#Cu beads, the copper is dispersed unevenly, which may result from the uneven access of the reducing agent to the Cu_2_O precursor. The differences in the distribution of copper in both types of cation exchangers may result from their different structure: macroporous or gel-type.

### 3.2. Attempt to Obtain G/Na#Cu, M/Na#Cu

In the preparative part of this study, reference should be made to the possibility of obtaining metallic copper-doped carboxylic cation exchangers with functional groups in the Na^+^ form. Such host materials show extraordinary thermal resistance, up to 450 °C, which is unique among ion exchangers as they are generally not thermally resistant. As the transformation of G/Na#Cu_2_O to G/Na#Cu and M/Na#Cu_2_O to M/Na#Cu by the sodium ascorbate proved ineffective, the order of transformations was changed. First, G/H#Cu was obtained, and then it was reacted with 1.0 M NaOH to transform –COOH groups to –COONa. When the transformation was carried out in a column, as in the case of pure resin, the beads swelled so much that the influent stopped flowing through the bed. Then, an attempt was made to achieve the desired transformation using the batch method. When G/H#Cu (1.0 g) was placed in an Erlenmeyer flask and shaken with 1.0 M NaOH solution, a fine, brown precipitate appeared in the water phase. Metallic copper moved from the resin phase to the water phase due to the swelling of the ion exchanger and the loosening of the polymeric porous skeleton. The precipitate after the reaction was separated, washed, dried and weighed. It turned out that almost 70.0 mg of the Cu, i.e., about 35% of its initial content, was not present in the examined sample (the fine precipitate remained brown during the reaction, separation and drying).

### 3.3. Thermogravimetric Studies, G/H vs. G/H#Cu, M/H vs. M/H#Cu

**Figure 5:**
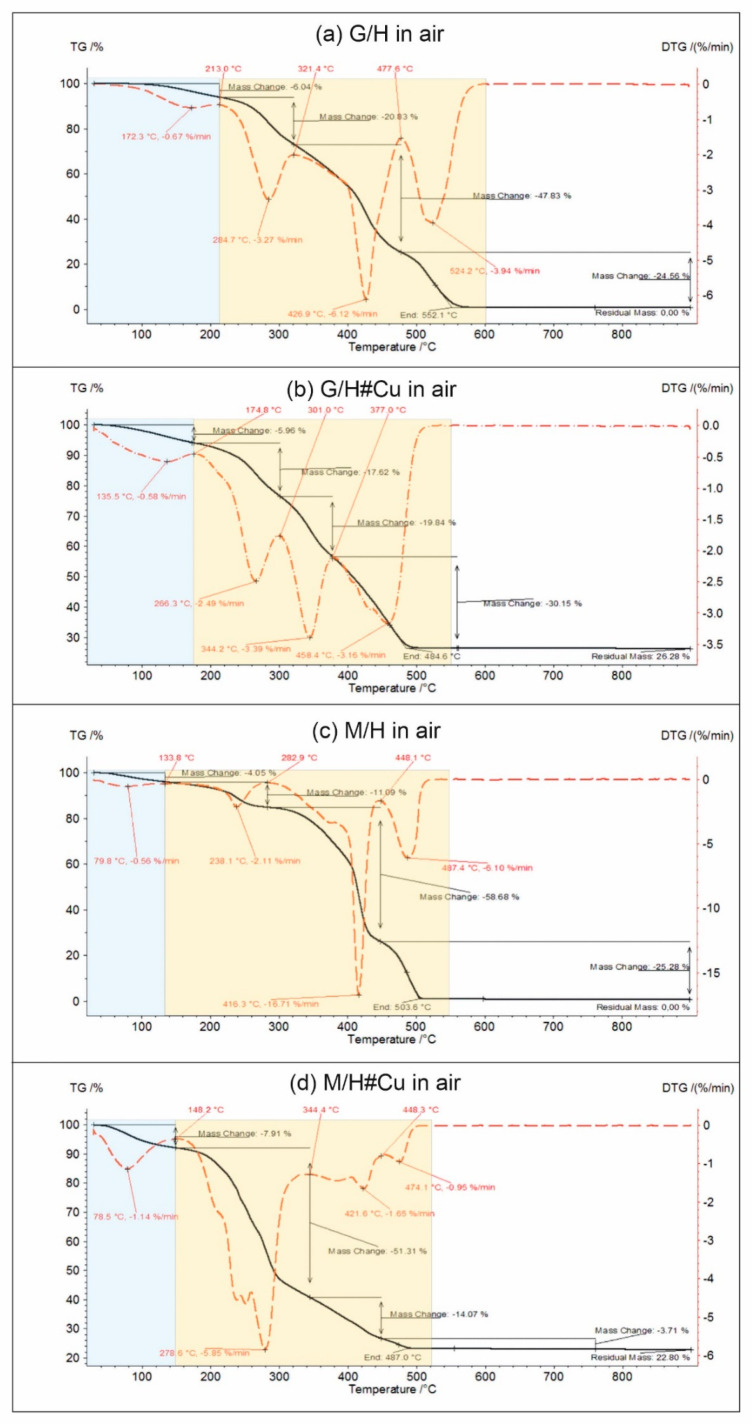
TG/DTG curves in air of G/H (**a**), G/H#Cu (**b**), M/H (**c**), M/H#Cu (**d**), Blue—dehydration, Yellow—polymeric matter decomposition.

**Figure 6:**
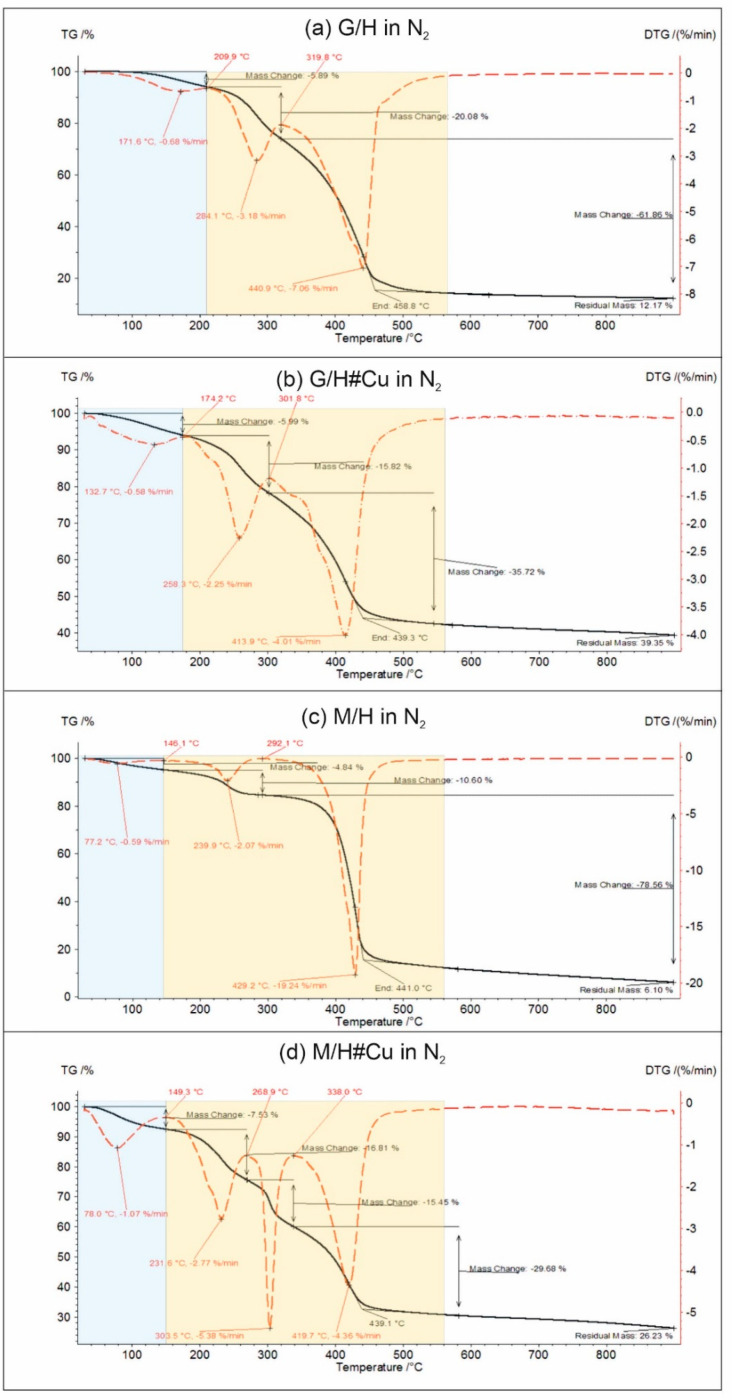
TG/DTG curves in N_2_ of G/H (**a**), G/H#Cu (**b**), M/H (**c**), M/H#Cu (**d**), Blue—dehydration, Yellow—polymeric matter decomposition.

**Figure 7:**
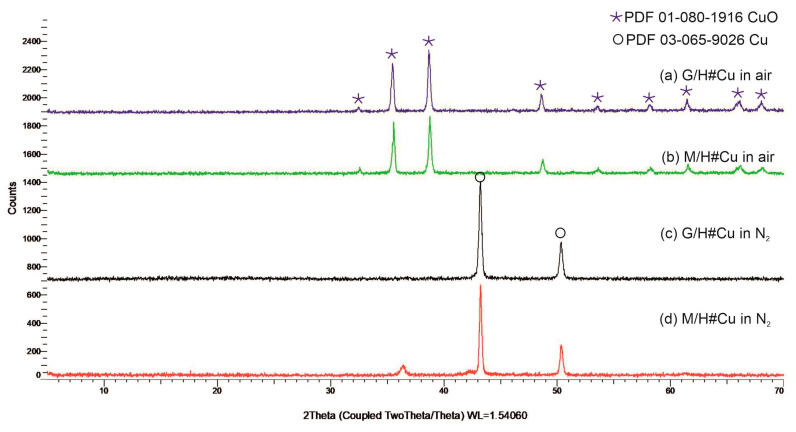
XRD patterns of solid residues after thermal analysis in air and in N_2_.

**Figure 8:**
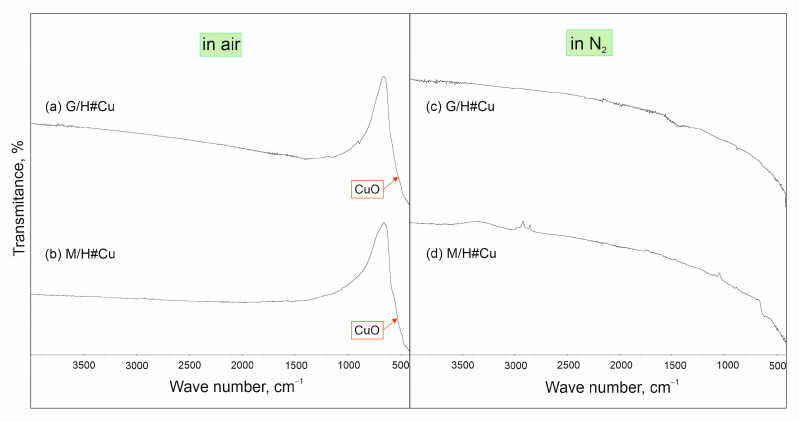
FTIR spectra of solid residues after thermal analysis in air and in N_2_.

Thermal analysis, including thermogravimetry (TG) and derivative thermogravimetry (DTG), is a useful tool for assessing the impact of metallic copper deposits on the decomposition of the polymeric phase during controlled heating up to 950 °C, either in oxidizing conditions (combustion in air) or in an inert atmosphere (pyrolysis under N_2_). The newly obtained G/H#Cu and M/H#Cu were subjected to thermal analysis, and their TG/DTG curves were compared with the ones of the starting materials (G/H and M/H) in analogous conditions. All samples subjected to thermal analysis were dried at 40 °C, i.e., in relatively mild conditions, so as to compare the results of experiments involving other thermally sensitive ion exchangers. The results are grouped according to the structure of the polymeric support (gel-type or macroporous) and the medium in which the process proceeded (air or N_2_). Figure 5 and Figure 6 show the TG/DTG curves of eight experiments with numerical data characterizing the particular transformation, end temperature and residual mass. The XRD patterns and FTIR spectra of the solid residues are presented in Figure 7 and Figure 8.

The thermal decomposition of G/H and M/H was previously analyzed in detail, concluding that in an air atmosphere it proceeded in four stages associated with growing mass losses, namely water evaporation, condensation of carboxylic groups to anhydrides, splitting of the previously dehydrated functional groups, and finally the ultimate combustion of the polymeric skeleton [34,42,43,44,45,46,47]. A significant difference in the thermal decomposition of G/H (Figure 5a) compared to M/H (Figure 5c) can be observed at 200–300 °C. Carboxyl groups attached to a skeleton of different structures (with polymer chains located close to each other or separated by macropores in the macromolecule) decomposed in different ways, including intermolecular dehydration of neighboring groups (with succinic anhydride type and glutaric anhydride type formation) and intramolecular dehydration of opposite located groups (with isobutyric anhydride type formation). In the case of G/H, a mass loss of as much as 20.0% resulted from both intermolecular and intramolecular dehydration due to the compact structure of the dry gel-type matrix and the proximity of polymer chains. In the case of M/H, the mass loss was much smaller, at 11.0%, probably because dehydration only occurred of carboxyl groups located next to each other.

Table 2 collates the data of all the thermal experiments to facilitate the comparison of the results. The data in the second column refer to the elimination of water, which is osmotic water molecules existing in the pores of resin and hydrogen-bonded water from hydration sites of carboxylic groups. The carboxylic cation exchangers in the H^+^ form (with undissociated functional groups) are weakly hydrated, regardless of the structure of the polymeric skeleton, and contain as little as 6.0% (G/H) and 4.0% (M/H) of water, which matches the first mass loss on the corresponding TG/DTG curves. The introduction of metallic copper into the matrix of G/H induced almost no change in the water content; G/H#Cu was as poor in water as G/H. Materials that act as water repellents are valuable and rare among ion exchangers. A feature such as hydrophobicity is desirable in various applications, for example in the case of antimicrobial action (metallic copper surfaces have been used to prevent bacterial growth, and the placement of fine copper particles within the water-repelling polymeric porous support can strengthen this action). The introduction of metallic copper to the matrix of M/H significantly increased the water content, from 4.0% (M/Cu) to almost 8.0% (M/H#Cu). This result corresponds to the interpretation of the reasons for the unexpected appearance of M/H#Cu because the carboxyl groups in the salt form are hydrated (as already mentioned, the blue color of M/H#Cu could have resulted from the surface oxidation of copper and the binding of Cu^2+^ ions by carboxyl groups). Moreover, when observing the first stage of the thermal analysis of the examined materials (Figure 5a–d), it can be seen that the temperature range of the dehydration processes was not the same. Both in an air atmosphere and under N_2_, dehydration of M/H and M/H#Cu proceeded up to about 150 °C, while in the case of G/H and G/H#Cu, it ended only at 200 °C (a gel-type polymeric matrix, strongly shrunken in the dry state, releases residual water with difficulty).

The data in Table 2 (part (a), third column) show that in an air atmosphere, the presence of copper in the sample shifted the polymeric matter decomposition towards a lower temperature. Subsequent peaks related to G/H#Cu and M/H#Cu decomposition (expressing the temperature of maximum decomposition rate) are recorded at temperatures significantly lower compared to G/H and M/H. The greatest acceleration of the decomposition process can be observed by comparing the TG/DTG curves of M/H (Figure 5c) and M/H#Cu (Figure 5d). Up to 300 °C, the mass loss in the case of M/H was only 15%, while in the case of M/H#Cu, it was 55%. In the temperature range of 220–300 °C, an intense transformation takes place in which two broad overlapping peaks can be observed, indicating decomposition of functional groups, and, in particular, very fast decarboxylation (at a temperature of about 120–150 °C lower than M/H). A similar trend can be observed in the case of the end temperature (Table 2, part (a), fourth column), as the G/H#Cu decomposition ended at a temperature nearly 70 °C lower compared to G/H. From data showing the residue left at 950 °C after thermal analysis in air (Table 2, part (a), last column), it can be seen that G/H and M/H burned completely, without forming ash. It can be assumed that the mass of residues after combustion of G/H#Cu (26.28%) and M/H#Cu (22.80%) resulted from the presence of metallic copper in both examined composite materials. An XRD analysis showed the formation of tenorite (CuO) to be present in the solid residue as a result of copper oxidation in the air atmosphere (Figure 7a,b). Moreover, in the FTIR spectra of both solid residues (Figure 8a,b), a broad band can be seen in the far region in the range 600–450 cm^−1^, which can be assigned to Cu(II)-O stretching vibrations. Taking into account the masses of both residues and their likely homogeneous composition (CuO was the only component), it was calculated that G/H#Cu contained approximately 21.0% Cu before the combustion process, while M/H#Cu had 18.2% Cu. These values confirm that Cu(I) in G/Na#Cu_2_O almost entirely transformed into Cu^0^ in G/H#Cu, while in the case of the pair M/Na#Cu_2_O and M/H#Cu, the transformation took place with an efficiency of the order of 90–95%. It should be taken into account that HIXs with Cu_2_O are highly hydrated (Table 1), while HIXs with Cu^0^ are weakly hydrated, which affects the copper content per unit mass of the sample.

Figure 6a–d show the results of thermal analysis of the four examined materials under N_2_. In an inert gas atmosphere, G/H and M/H decomposed differently than under air, and on the TG/DTG curves there are only three separate transformations involving water evaporation, one-step splitting of functional groups, and decomposition of the polymeric matrix (Figure 6a,c). During pyrolysis of such materials, a characteristic solid residue is formed (carbonisate, carbon), which is a mixture of various high molecular organic compounds. The residual mass after pyrolysis of G/H (12.15%) was twice as much as the residual mass after pyrolysis of M/H (6.10%), which, given the same chemical composition of both resins, resulted above all from differences in their internal structure (the more compact gel-type skeleton without real pores limited the flow of gaseous matter (to and from the interior of the beads), whereas the more open macroporous skeleton was easily accessible to gaseous matter). It should be added that relatively, pyrolysis of CCEs does not produce a large amount of carbon, because their polymeric matter, due to the presence of carboxylic groups, contains as much as 35.0% oxygen.

The data in Table 2, part (b), show that under N_2_ the presence of metallic copper in the sample shifted the polymeric matter decomposition towards a lower temperature, with this effect more noticeable in the case of the M/H and M/H#Cu pair than G/H and G/H#Cu. Up to 350 °C, the mass loss for M/H was about 18.0%, whereas for M/H#Cu it was above 45.0% (in Figure 7d, compared to Figure 7c, a new peak can be seen with a maximum decomposition rate at 303.5 °C related to the ultimate destruction of the carboxylic groups).

We have previously stated that in the case of pyrolysis of CCEs containing CuO and Cu_2_O, the most interesting issue concerns the solid residue (pyrolysate), the amount and composition of which was the result of the interaction of organic and inorganic matter in the oxidation–reduction reactions. The solid residue consisted of carbon (the product of polymeric matter decomposition without access to air) and chemically transformed inorganic matter (for example, the CuO contained in the HIX was reduced during pyrolysis to Cu^0^ identified in the solid residue). As hydrogen was needed to reduce CuO to Cu^0^ (formed as a result of the thermal decay of the hydrocarbon chain from which the polymeric skeleton is built), we considered its consumption for this purpose to be the cause of the formation of the additional amount of carbon (more than the amount resulting from pyrolysis of pure resin). In this study, thermal decomposition of G/H#Cu and M/H#Cu resulted in a solid residue consisting of carbon and metallic copper (Figure 7c,d). In this case, however, the reduction did not take place because metallic copper was already included in the starting material. Therefore, it was interesting to analyze the results of pyrolysis, during which the inorganic matter did not change its form. It turned out that in the case of G/H#Cu and M/H#Cu, the mass of the solid residue after pyrolysis was greater than the sum of the mass of the solid residue after pyrolysis of pure resin and the mass of copper in the sample undergoing pyrolysis (for the G/H and G/H#Cu pair, it was 39.35% > 12.17% + 21.0%; for the M/H and M/H#Cu pair, it was 26.23% > 6.10% + 18.24%). These calculations show that in the case of G/H#Cu, an additional 6.0% of carbon was formed, and in the case of M/H#Cu, about 2.0% was formed (the carboxylic cation exchanger gel-type structure, with narrow pores filled or maybe even blocked by metallic copper, underwent thermal transformation with more difficulty, which favored the reactions of carbonaceous matter condensation).

Pyrolysis of HIXs, in contrast to combustion, is of practical importance as it leads to further useful composite materials consisting of carbonaceous and inorganic matter. Pyrolysates obtained on the basis of CCEs have a unique composition thanks to the high content of functional groups and consequently low molecular mass. They contain a relatively large amount of inorganic matter in relation to organic matter (carbon). As a result of G/H#Cu pyrolysis, a solid residue was created, including 50% metallic copper and 50% carbon.

### 3.4. Thermal Analysis as a Useful Method for Evaluating CuO-, Cu_2_O-, Cu^0^-Doped Carboxylic Cation Exchangers

**Scheme 2:**
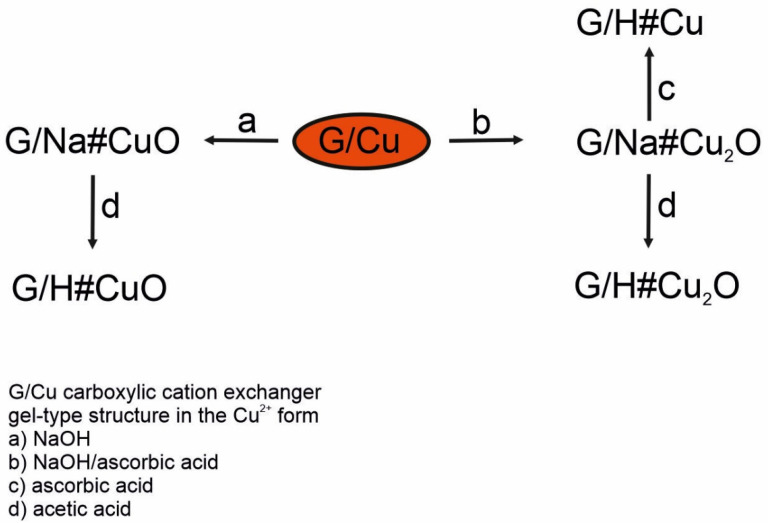
Synthesis of HIXs containing CuO, Cu_2_O and Cu^0^ deposit within the matrix of a gel-type carboxylic cation exchanger.

**Scheme 3:**
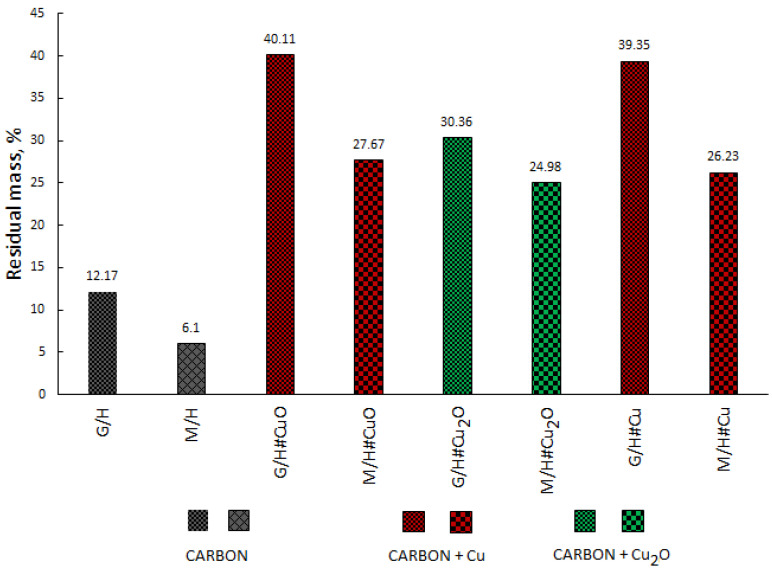
Solid residues after pyrolysis of several copper-containing composites in comparison to pure CCEs [34,35].

In this study, as well as in our previous two works [34,35], we have described a family of hybrid ion exchangers, which are CuO-, Cu_2_O- and Cu^0^-doped carboxylic cation exchangers of both structures. This group consists of several products (Scheme 2), whose properties are significantly influenced not only by the kind of inorganic deposit but also the resin structure and the ionic form of the carboxylic groups (H^+,^ Na^+^, Cu^2+^). Thermal analysis proved to be a convenient method of evaluating these materials as it provided information on their various interesting properties, not only related to their thermal behavior. The first step of thermal analysis, dehydration, showed that some of the materials uniquely contained little bound water (G/H#Cu, 5.96%), while others were hydrated to a very high degree (M/Na#Cu_2_O, 21.35%). The TG/DTG curves showed that dehydration took place in different temperature ranges, even up to 300 °C, and that among the obtained composites were materials resistant to temperatures of 450 °C (especially when the polymeric host materials contained the carboxylic group in the Na^+^ form). Thermal analysis in air showed that the dispersion of CuO, Cu_2_O and Cu^0^ accelerated the decomposition of the polymeric phase to varying degrees. When the functional groups of CCEs were in the H^+^ form, regardless of the composition of the inorganic phase at the start, the residue after combustion was pure CuO. On the basis of its content, the elemental copper content in the samples subjected to thermal analysis was calculated (Table 3, last row). As shown by the data in Table 3, the transformation of Cu^2+^ ions bonded by the functional groups into CuO, Cu_2_O and Cu^0^ occurred almost entirely, while the transformation of the M/H#Cu_2_O to M/H#Cu^0^ occurred with a slightly lower efficiency. In the case of thermal analysis under N_2_, the most interesting issue was the mass of the solid residue and the proportion of carbonaceous and inorganic components. Evaluating the pyrolysates in terms of mass and components (Scheme 3), the impact of the structure of the host material on the mass of solid residue can be seen, as well as the impact of the inorganic phase on the amount of carbon (pyrolysis of composite materials gave relatively more carbon than pyrolysis of pure resin) and the impact of the polymeric phase on the reduction (if possible) of the inorganic phase (during pyrolysis of G/H#Cu_2_O and M/H#Cu_2_O, reduction of Cu_2_O to Cu^0^ did not occur, but it occurred during pyrolysis of G/Na#Cu_2_O and M/Na#Cu_2_O).

The presented hybrid ion exchangers are a group of composite materials with a very high copper content, which can be obtained under mild conditions as a result of simple transformations using available and cheap reagents. It can be expected that their characteristic feature will be broadly understood to be their multifunctionality, resulting from the content of copper in different oxidation states (which affects the sorption, oxidation, reduction, deoxidation and antimicrobial properties), from the different ionic forms of carboxylic groups (low vs. high thermal stability of the host material, hydrophilicity vs. hydrophobicity of the host material, swelling in water vs. shrinkage in water of the host material), and from the different structure of the host material (compact gel-type structure vs. open macroporous structure).

## 4. Conclusions

In ambient conditions, using ascorbic acid as a green reducing agent, it was shown to be possible to obtain composite materials belonging to the groups of hybrid ion exchangers, which contained even more than 20.0 wt% metallic copper dispersed within the matrix of carboxylic cation exchangers, of both the gel-type and with a macroporous structure. Metallic copper particles were embedded within the matrix of the resins thanks to the reduction of Cu_2_O particles precipitated there earlier. It was possible to introduce a theoretically possible amount of Cu^0^ into a gel-type polymeric carrier when ascorbic acid acted as reducer of Cu_2_O and a reagent transforming the functional groups from Na^+^ in the H^+^ form. In this product (G/H#Cu), metallic copper particles were tightly surrounded by compact polymeric matter exhibiting a limited affinity for water, which protected the copper from oxidation. Thus, without the use of special conditions (inert atmosphere, non-aqueous media), it was possible to obtain stable materials despite the tendency of copper to oxidize. When a macroporous carboxylic cation exchanger was used as the host material, metallic copper particles were situated inside the polymeric beads, while Cu^2+^ ions bonded by carboxylic groups appeared on their surface. Attempts to obtain composites such as metallic copper inside a carboxylic cation exchanger in the Na^+^ form (these would be thermally resistant HIXs) were not successful, as the resin in the Na^+^ form swelled so strongly that, from its interior, Cu^0^ particles in significant quantities passed into the aqueous phase. Thermal analysis showed that G/H#Cu contained 6.0% bound water, less than M/H#Cu (7.50%), and that the metallic copper dispersed within the resin matrix accelerated its decomposition in both media (M/H#Cu decomposed faster than G/H#Cu). The solid residue after combustion of G/H#Cu and M/H#Cu was CuO (26.28% and 22.80%), and after pyrolysis the obtained solid residue (39.35% and 26.23%) was a mixture of carbon (50%) and metallic copper (50%). The results of this study complement our previous studies performed on carboxylic cation exchangers with CuO and Cu_2_O deposits.

## Figures and Tables

**Table 1:** Substrates’ characterization.

Code	Cu, mg/g	Water, % *
G/Cu	228.9	10.22
M/Cu	217.9	9.37
G/Na#Cu_2_O	199.2	15.96
M/Na#Cu_2_O	180.3	21.35

* hygroscopic/bounded water content determined by thermogravimetry [34,35].

**Table 2:** Statement of thermal decomposition results.

Sample Code	Water Mass Loss, wt%	Peak Temperature of Polymer Decomposition, °C	End Temperature, °C	Residual Mass at 900 °C, wt%
(a) Decomposition in air				
G/H	6.04	284.7, 426.9, 524.2	552.1	0.00
G/H#Cu	5.96	266.3, 344.2, 458.4	484.6	26.28
M/H	4.05	238.1, 416.3, 487.4	503.6	0.00
M/H#Cu	7.91	230.6, 312.4, 407.0	487.0	22.80
(b) Decomposition in N_2_				
G/H	5.89	284.1, 440.9	458.8	12.17
G/H#Cu	5.99	258.3, 413.9	439.3	39.35
M/H	4.84	239.9, 429.2	441.0	6.10
M/H#Cu	7.53	231.6, 303.5, 417.7	439.1	26.23

**Table 3:** Residual mass after combustion of selected CCEs containing Cu^2+^, CuO, Cu_2_O and Cu^0^ [34,35].

Code	G/Cu	G/H#CuO	G/H#Cu_2_O	G/H#Cu	M/Cu	M/H#CuO	M/H#Cu_2_O	M/H#Cu
Residual mass (CuO), %	29.86	30.13	30.50	26.28	26.65	24.52	26.63	22.80
Cu in HIX, mg/g	238.9	240.1	244.0	210.2	213.2	196.1	213.0	182.4

## Data Availability

Data will be made available on request.
